# Identification of Three Distinct Subgroups in Antiphospholipid Syndrome: Implication for Sex Differences and Prognostic Outcomes from a Multicenter Study

**DOI:** 10.1002/advs.202415291

**Published:** 2025-02-18

**Authors:** Chen Chen, Ao Zhang, Jianhui Cheng, Zhongqiang Yao, Juan Meng, Yilu Qin, Qingyi Lu, Yufei Li, Xiangjun Liu, Tianhao Li, Chao Hou, Yundi Tang, Hongjiang Liu, Ning Xu, Sai Dong, Xinxin Li, Fangmin Xu, Jianping Guo, Chun Li

**Affiliations:** ^1^ Department of Rheumatology and Immunology Peking University People's Hospital Beijing 100044 China; ^2^ School of Information and Communication Engineering Beijing University of Posts and Telecommunications Beijing 100876 China; ^3^ State Key Laboratory of Neurology and Oncology Drug Development Nanjing Jiangsu 210023 China; ^4^ Department of Rheumatology and Immunology Peking University Third Hospital Beijing 100191 China; ^5^ Department of Rheumatology and Immunology Beijing Chaoyang Hospital Affiliated to Capital Medical University Beijing 100020 China; ^6^ Department of Rheumatology and Immunology Affiliated Xinxiang Central Hospital of Xinxiang Medical University Xinxiang Henan 453000 China; ^7^ Department of Biomedical Informatics School of Basic Medical Sciences Peking University Health Science Center Beijing 100191 China; ^8^ Department of Rheumatology and Immunology West China Hospital Sichuan University Chengdu Sichuan 610041 China

**Keywords:** antiphospholipid syndrome, prognosis, proteomics, sex, subphenotypes

## Abstract

Antiphospholipid syndrome (APS) is a heterogeneous autoimmune disease with persistent antiphospholipid antibodies. This study aimed to identify unrecognized APS subgroups from multicenter cohorts (*n* = 760, training: *n* = 415; validation: *n* = 345). Patients are stratified through unsupervised K‐means clustering analysis. Prognostic outcomes are evaluated using Kaplan‐Meier survival analyses. Proteomic analysis is conducted on primary APS patients (*n* = 36) and healthy controls (*n* = 12). Key molecule insulin‐like growth factor 1 is validated using ELISA. Three clusters are identified. Cluster 1 (*n* = 320, 42.1%) is completely consisted of females (100%), with predominant occurrence of pregnancy morbidity (88.8%) but low incidences of thrombocytopenia (18.4%) and thrombosis (15.0%), and a favorable prognosis. Cluster 2 (*n* = 309, 40.7%) is predominantly female (99.4%) and characterized by high thrombosis (85.8%) and thrombocytopenia (46.6%), low pregnancy morbidity (13.6%), and poor prognosis. Cluster 3 (*n* = 131, 17.2%) is predominantly male (99.2%), exhibiting highest thrombosis (96.2%) and moderate thrombocytopenia (32.8%), with worst prognosis. Immunological and proteomic analyses clearly differentiated three clusters. This study reveals a distinct difference between obstetric and thrombotic APS, and a sex‐based distinction within thrombotic APS. Three APS subgroups display unique clinical and molecular characteristics, and marked difference in prognostic outcomes.

## Graphical abstract

A large‐scale, multicenter, and two‐stage study is conducted to define antiphospholipid syndrome (APS) subgroups. By applying unsupervised K‐means clustering algorithm, the authors identified three distinct APS subgroups: obstetric APS, female thrombotic APS (tAPS), and male tAPS, which display marked differences in clinical manifestations and prognosis. Insulin‐like growth factor 1 is identified as key molecule linked to poor prognosis in male tAPS.

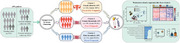

## 1 Introduction

Antiphospholipid syndrome (APS) is a rare autoimmune disease characterized by the presence of antiphospholipid antibodies (aPLs), which causes thrombosis and/or pregnancy morbidity. The annual incidence of APS is 2.1 per 100 000 population, and the estimated prevalence is 50 per 100 000 population.^[^ ^1^ ^]^ The male‐to‐female ratio in APS is 1:5.^[^ ^2^ ^]^ The prevalence of aPLs at a single time point is 14.5%,^[^ ^3^ ^]^ and it is also high among young patients with thrombosis and pregnancy morbidity.^[^ ^4^ ^]^ APS can occur independently, known as primary APS, or complicate with other autoimmune diseases, namely secondary APS.^[^ ^2^, ^5^, ^6^ ^]^ Nowadays, it's generally accepted that APS could also be categorized into either thrombotic APS (tAPS) or obstetric APS (oAPS) based on their distinct clinical features.^[^ ^7^ ^]^ Substantial differences may exist in the pathogenesis between tAPS and oAPS despite their shared autoantibody profiles.^[^ ^7^ ^]^ Clinical heterogeneity is also observed within tAPS patients.^[^ ^8^ ^]^ However, so far, few studies have focused on sex differences within tAPS patients.

Previously, several studies have applied cluster analyses to identify subgroups among aPL‐positive or ANA‐positive patients with APS.^[^ ^9^, ^10^, ^11^, ^12^, ^13^ ^]^ Notably, all of these studies have applied hierarchical cluster analysis, and most of them are single‐center studies without validation. None of the reports further investigates the possible molecular mechanism(s) involved in the pathogenesis of different APS subgroups. In the present study, we conduct a large‐scale, multicenter, and two‐stage (training and validation) study to define the APS subgroups. In addition, we apply an unsupervised K‐means clustering algorithm to stratify the study subjects according to multiple clinical and laboratory indicators. K‐means is a powerful clustering algorithm in machine learning to identify clusters by distinguishing the data points in large datasets. The K‐means algorithm assigns the observations to the nearest cluster center based on their similarity and capable of further validating other datasets. In contrast, hierarchical clustering relies on the intrinsic hierarchical structure of the data, typically requiring a one‐time processing of the entire dataset to construct the clustering tree. This feature reduces its flexibility and adaptability when handling a validation cohort.^[^ ^14^ ^]^ K‐means clustering analysis has been widely used to stratify heterogeneous diseases into “homogeneous” subgroups.^[^ ^15^, ^16^, ^17^, ^18^ ^]^ Finally, we perform a proteomic analysis to identify key molecule(s) and reveal the potential mechanism(s) underlying the pathogenesis of the distinct APS subgroups.

## 2 Results

### 2.1 By Applying the Unsupervised K‐means Clustering Analysis, Three Distinct APS Subgroups Were Identified

**Figure 1:**
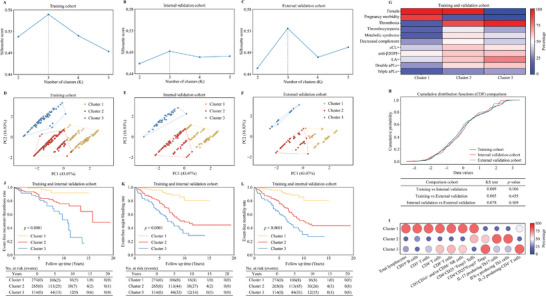
Cluster analysis and main characteristics. An optimal number of clusters (K = 3) was fitted best for all three cohorts: A) in training cohort (*n* = 415, silhouette coefficient = 0.57). B) In internal validation cohort (*n* = 238, silhouette coefficient = 0.49). C) In external validation cohort (*n* = 107, silhouette coefficient = 0.54). D) PCA scatter plot for the training cohort. E,F) The model developed from the training cohort was applied to both the internal and external validation cohorts. G) Heat map showing main characteristics in all cohorts (*n* = 760). H) CDF was compared by the KS test to determine whether the distribution was different between cohorts. The values of KS test range from 0 to 1, with a lower value indicating better similarity. The *p* values also demonstrated there was no significant difference in the compared cohorts (*p*>0.05). I) Bubble plots showing the ratio of each cluster's median to the overall median as the relative percentage of immune cell subpopulations and molecules in naïve APS patients. Total lymphocytes, CD19^+^B cells, CD3^+^T cells, CD4^+^T cells, CD8^+^T cells, and CD3^−^CD16^+^ and/or CD56^+^ NK cells (n = 69); CD4^+^CD25^−/+^Foxp3^−^Teffs and CD4^+^CD25^hi^Foxp3^+^ Tregs (*n* = 67); IL‐17 producing Th17 cells, IFN‐γ producing Th1 cells, and IL‐2 producing CD4^+^ T cells (*n* = 36). Dot size and color indicate the relative expression strength levels. Chi‐square test was used for assessing difference between the proportions. J–L) Kaplan–Meier analysis of cumulative event‐free survival in APS patients and the *p* values were calculated with the log‐rank test, which showed recurrent thrombosis, major bleeding, and mortality event‐free survival in the training and internal validation cohort. The numbers below the figures denoted the number of patients at risk in each cluster.

First, we applied the unsupervised K‐means clustering algorithm in the training cohort. As shown in **Figure** **1**A, the optimal number of clusters (K) was set to be three (K = 3) with a silhouette score of 0.57 in the training cohort. The optimal number of clusters (K = 3) was also fitted best for both internal validation cohort (silhouette score = 0.49) and external validation cohort (silhouette score = 0.54) (Figure 1B,C). Notably, by the principal component analysis (PCA), the APS patients were also clearly separated into three clusters in three independent cohorts (Figure 1D–F). The two principal components explained 43.07% and 16.93% of the total variance, respectively. On principal component 1, the coefficients of “pregnancy morbidity,” “thrombotic events,” and “thrombocytopenia, hemolytic anemia, immunoglobulin, and complement abnormalities” were 0.53, 0.51, and 0.38, respectively, while “sex,” “age,” and “other clinical indicators” had the weight coefficients of 0.75, 0.48, and 0.27 on principal component 2. The greater the weight coefficient, the greater the contribution. This indicated that the two principal components retain most of the information from the original data. According to the main indices in Table S1 (Supporting Information), these APS patients were also distinguished into three clusters and were visualized in the clustering diagrams (Shown in Figure 1G; Figure S1A–C, Supporting Information). Furthermore, cumulative distribution functions (CDF) was analyzed by the Kolmogorov Smirnov (KS) test to confirm that the distributions of the training and two validation sets were highly consistent, and there were no significant differences between three cohorts (overall KS value < 0.078, *p* > 0.106) (Figure 1H).

### 2.2 Cluster 1: “Female Obstetric APS”

Cluster 1 (*n* = 320, 42.1%) was entirely consisted of female patients (100%) with a younger age of onset (33 years, interquartile range, IQR 31–37 years). This cluster had the highest incidence of pregnancy morbidity (88.8%) and primary APS (70.6%) but a low incidence of thrombocytopenia (18.4%), thrombosis (15.0%), and metabolic syndrome (15.0%). In addition, this cluster showed relatively low incidences of positivity to three aPLs: aCL (40.6%), anti‐β2GPI (53.1%), LA (43.8%), double aPLs (30.0%) and triple aPLs (15.0%). Similar pattern was seen in training and validation cohorts (shown in Figure S1, Supporting Information).

### 2.3 Cluster 2: “Female Thrombotic APS”

Cluster 2 (*n* = 309, 40.7%) was also predominantly consisted of female patients (99.4%) but with more advanced age of onset (50 years, IQR 36–62 years). This cluster displayed a relatively high incidence of thrombosis (85.8%) and a low incidence of pregnancy morbidity (13.6%). In addition, the patients from this cluster showed a high incidence of thrombocytopenia (46.6%), metabolic syndrome (54.7%), and less primary APS (43.7%). This cluster also had higher rates of decreased complements (52.1%) and elevated Ig levels (38.2%). Compared to the Cluster 1, this cluster displayed a higher incidence of positivity to aPLs: aCL (59.9%), anti‐β2GPI (60.5%), LA (60.8%), double aPLs (56.6%), and triple aPLs (27.5%).

### 2.4 Cluster 3: “Male Thrombotic APS”

Cluster 3 (*n* = 131, 17.2%) was primarily consisted of male patients (99.2%) with a median age of onset (41 years, IQR 33–56 years). This cluster displayed the highest incidence of thrombosis (96.2%), LA positivity (74.8%), double aPLs (60.3%), and triple positivity rate of aPLs (31.3%). More than half of patients were primary APS (58.8%). In addition, this cluster had a moderate metabolic syndrome (42.0%), decreased complement (40.5%), and increased smokers (19.1%). There was no significant difference between the three independent cohorts. Clinical and laboratory characteristics of the study cohorts were detailed in **Table** **1** (combined cohort) and Tables S4 and S5 (Supporting Information) (three independent cohorts).

**Table 1:** Characteristics of three APS clusters in combined training and validation cohorts.

	Cluster 1 [*n* = 320, 42.1%]	Cluster 2 [*n* = 309, 40.7%]	Cluster 3 [*n* = 131, 17.2%]	*p‐*value
Female, *n* (%)	320 (100%)	307 (99.4%)	1 (0.8%)	<0.001
Age, years, median (IQR)	33 (31‐37)	50 (36‐62)	41 (33‐56)	<0.001
aGAPSS, median (IQR)	5 (4‐9)	9 (5‐13)	9 (5‐13)	<0.001
Pregnancy morbidity, *n* (%)	284 (88.8%)	42 (13.6%)	0 (0%)	<0.001
Thrombosis, *n* (%)	48 (15.0%)	265 (85.8%)	126 (96.2%)	<0.001
Arterial thrombosis, *n* (%)	25 (7.8%)	157 (50.8%)	73 (55.7%)	<0.001
Venous thrombosis, *n* (%)	30 (9.4%)	156 (50.5%)	73 (55.7%)	<0.001
Thrombocytopenia, *n* (%)	59 (18.4%)	144 (46.6%)	43 (32.8%)	<0.001
Hemolytic anemia (HA), *n* (%)	11 (3.4%)	45 (14.6%)	9 (6.9%)	<0.001
Neuropsychiatric disorders, *n* (%)	2 (0.6%)	42 (13.6%)	9 (6.9%)	<0.001
Smoking, *n* (%)	18 (5.6%)	22 (7.1%)	25 (19.1%)	<0.001
Primary APS, *n* (%)	226 (70.6%)	135 (43.7%)	77 (58.8%)	<0.001
Metabolic syndrome, *n* (%)	48 (15.0%)	169 (54.7%)	55 (42.0%)	<0.001
Hypertension, *n* (%)	31 (9.7%)	122 (39.5%)	40 (30.5%)	<0.001
Hyperlipidemia, *n* (%)	15 (4.7%)	76 (24.6%)	25 (19.1%)	<0.001
Diabetes, *n* (%)	18 (5.6%)	50 (16.2%)	13 (9.9%)	<0.001
aCL positivity, *n* (%)	130 (40.6%)	185 (59.9%)	72 (55.0%)	<0.001
anti‐β2GPI positivity, *n* (%)	170 (53.1%)	187 (60.5%)	80 (61.1%)	0.227
LA positivity, *n* (%)	140 (43.8%)	188 (60.8%)	98 (74.8%)	<0.001
aCL and anti‐β2GPI positivity, *n* (%)	69 (21.6%)	132 (42.7%)	55 (42.0%)	<0.001
aCL and LA positivity, *n* (%)	65 (20.3%)	111 (35.9%)	51 (38.9%)	<0.001
anti‐β2GPI and LA positivity, *n* (%)	58 (18.1%)	102 (33.0%)	55 (42.0%)	<0.001
Double aPLs positivity, n (%)	96 (30.0%)	175 (56.6%)	79 (60.3%)	<0.001
Triple aPLs positivity, *n* (%)	48 (15.0%)	85 (27.5%)	41 (31.3%)	<0.001
Decreased complement, *n* (%)	95 (29.7%)	161 (52.1%)	53 (40.5%)	<0.001
Elevated Ig, *n* (%)	79 (24.7%)	118 (38.2%)	48 (36.6%)	0.002

IQR, interquartile range; aGAPSS, adjusted global anti‐phospholipid syndrome score; aPL, antiphospholipid antibody; LA, lupus anticoagulant; aCL, anticardiolipin antibody; anti‐β2GPI, anti‐β2 glycoprotein I antibody; C3, complement 3; C4, complement 4; Ig, immunoglobulin. Significance was assessed using one‐way ANOVA;.

### 2.5 Immunological Characteristics of the Three Clusters

Next, we analyzed immune cell subsets from the treatment‐naïve APS patients in three clusters. Cluster 1 had the highest number of total lymphocytes (Cluster 1 vs Cluster 2, *p* = 0.004; Cluster 1 vs Cluster 3, *p =* 0.016), CD19^+^ B cells (Cluster 1 vs Cluster 2, *p =* 0.019; Cluster 1 vs Cluster 3, *p =* 0.021), CD3^+^ T cells (Cluster 1 vs Cluster 2, *p =* 0.015; Cluster 1 vs Cluster 3, *p =* 0.054), CD8^+^ T cells (Cluster 1 vs Cluster 2, *P =* 0.004; Cluster 1 vs Cluster 3, *p =* 0.065), and the lowest number of IL‐17 producing Th17 cells (Cluster 1 vs Cluster 2, *p =* 0.005; Cluster 1 vs Cluster 3, *p =* 0.038). Cluster 2 exhibited the highest number of IFN‐γ producing Th1 cells (Cluster 2 vs Cluster 1, *p =* 0.065; Cluster 2 vs Cluster 3, *p =* 0.047), IL‐17 producing Th17 cells (Cluster 2 vs Cluster 1, *p =* 0.005; Cluster 2 vs Cluster 3, *p =* 0.361), and the lowest number of IL‐2 producing CD4^+^ T cells (Cluster 2 vs Cluster 1, *p =* 0.019; Cluster 2 vs Cluster 3, *p =* 0.128), CD8^+^ T cells (Cluster 2 vs Cluster 1, *p =* 0.004; Cluster 2 vs Cluster 3, *p =* 0.039), and CD3^−^CD16^+^ and/or CD56^+^ NK cells (Cluster 2 vs Cluster 1, *p =* 0.017; Cluster 2 vs Cluster 3, *p =* 0.142). Cluster 3 had the highest number of CD4^+^CD25^hi^Foxp3^+^ regulatory T cells (Tregs) (Cluster 3 vs Cluster 1, *p =* 0.054; Cluster 3 vs Cluster 2, *p =* 0.073) and the lowest number of CD4^+^CD25^−/+^Foxp3^−^ effector T cells (Teffs) (Cluster 3 vs Cluster 1, *p =* 0.010; Cluster 3 vs Cluster 2, *p =* 0.017) (shown in Figure 1I).

### 2.6 Cumulative Incidence Analysis for Recurrent Thrombosis, Major Bleeding, and Mortality in Three Clusters

Next, we assessed the cumulative incidences of event‐free recurrent thrombosis, major bleeding, and mortality in combined training and internal validation cohorts. As shown in Figure 1J–L, in general, the patients from Cluster 1 displayed the best prognosis, while the patients from Cluster 3 showed the worst prognosis. The 5‐year event‐free outcomes for recurrent thrombosis in the three clusters were 98.8%, 85.0%, and 82.3% (Cluster 1 vs Cluster 2, *p<*0.001; Cluster 1 vs Cluster 3, *p<*0.0001; Cluster 2 vs Cluster 3, *p* = 0.243), respectively, and the 10‐year recurrent thrombosis‐free rates were 91.7%, 75.5%, and 51.7% (Cluster 1 vs Cluster 2, *p<*0.0001; Cluster 1 vs Cluster 3, *p<*0.0001; Cluster 2 vs Cluster 3, *p =* 0.046). The 5‐year major bleeding‐free rates were 95.7%, 76.1%, and 61.9% (Cluster 1 vs Cluster 2, *p<*0.0001; Cluster 1 vs Cluster 3, *p<*0.0001; Cluster 2 vs Cluster 3, *p =* 0.006), and the 10‐year major bleeding‐free rates were 80.2%, 48.5%, and 31.9% (Cluster 1 vs Cluster 2, *p<*0.0001; Cluster 1 vs Cluster 3, *p<*0.0001; Cluster 2 vs Cluster 3, *p =* 0.010). The 5‐year cumulative survival free from mortality event were 95.7%, 75.8%, and 60.9% (Cluster 1 vs Cluster 2, *p<*0.0001; Cluster 1 vs Cluster 3, *p<*0.0001; Cluster 2 vs Cluster 3, *p =* 0.005), and the 10‐year survival rates were 80.2%, 49.0%, and 30.1% (Cluster 1 vs Cluster 2, *p<*0.0001; Cluster 1 vs Cluster 3, *p<*0.0001; Cluster 2 vs Cluster 3, *p =* 0.005). These observations were similar between the training cohort (Figure S2A‐C, Supporting Information) and the internal validation cohort (Figure S2D‐F, Supporting Information).

### 2.7 Proteomic Analysis for Primary APS Patients from Three Clusters

**Figure 2:**
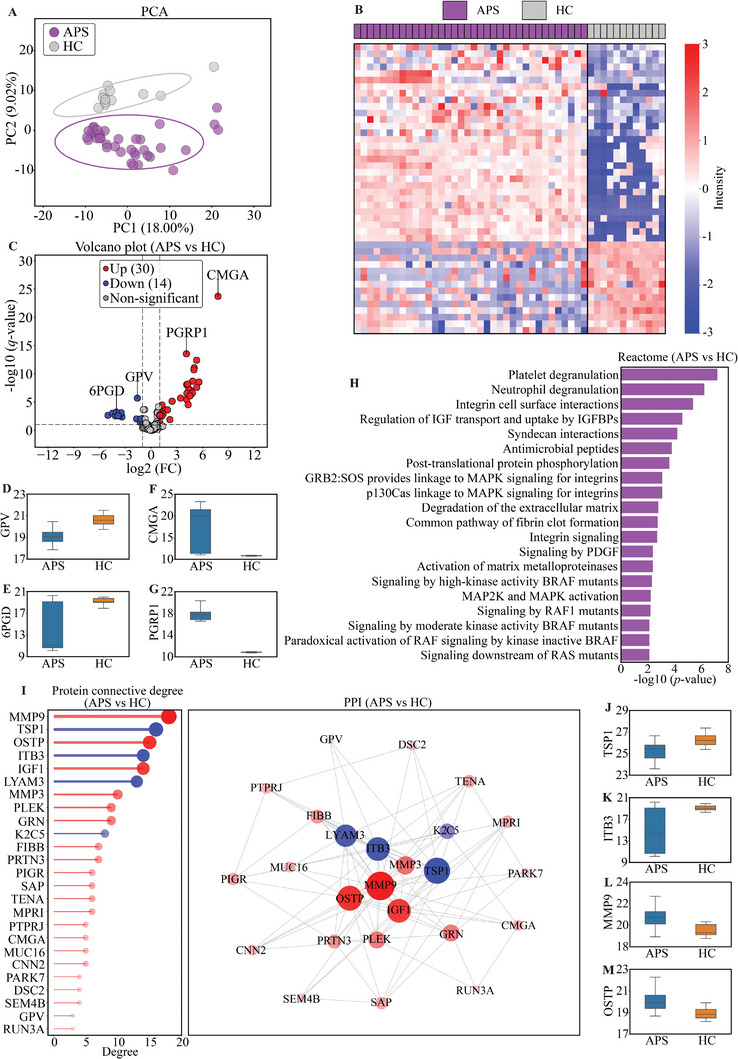
DEPs and bioinformatic analysis (APS patients vs HCs). A) PCA score plot for proteomic in APS (*n* = 36) versus HC (*n* = 12), the ellipse represents the 95% confidence interval. B) Heat map showing DEPs in APS patients versus HC. C) Volcano plot showing DEPs in APS versus HC. D–G) Box plots displaying the two most significantly downregulated (GPV, 6PGD) and upregulated (CMGA, PGRP1) proteins in APS versus HC, selected based on the highest negative log10‐transformed *q*‐values. H) Top 20 Reactome pathway enrichment results of DEPs in APS versus HC. I) The top 25 connection degree proteins and PPI network in APS versus HC. Proteins upregulated in APS were represented in red, while downregulated proteins were shown in blue. The size of the circle represented the magnitude of the connection. J–M) Box plots illustrating proteins with the highest connectivity degrees in APS versus HC, showing TSP1 and ITB3 as the most significantly downregulated and MMP9 and OSTP as the most upregulated.

To further elucidate the potential immunopathological mechanisms underlying the three APS subphenotypes, we conducted a serum proteomic analysis for 36 primary APS patients (*n* = 12 from each cluster) and 12 sex‐ and age‐matched healthy controls (HCs) to identify differentially expressed proteins (DEPs). As shown in **Figure** **2**A, by the PCA analysis, the APS patients and HCs were separated into two components. Using a *q*‐value of less than 0.1 and considering proteins with at least two fold changes (FCs), a total of 44 DEPs were identified. A volcano plot was constructed to visualize the 44 DEPs (30 upregulated, 14 downregulated) between APS and HC (Figure 2B). The key DEPs and their expression were shown in Figure 2C–G, including platelet glycoprotein V (GPV, FC = 0.33, *q*‐value = 2.10 × 10^−6^), 6‐phosphogluconate dehydrogenase (6PGD, FC = 0.06, *q*‐value = 6.19×10^−4^), chromogranin‐A (CMGA, FC = 219.82, *q*‐value = 2.02×10^−24^), and peptidoglycan recognition protein 1 (PGRP1, FC = 17.22, *q*‐value = 3.01 × 10^−14^). Top 20 biological processes were identified by the Reactome analysis based on the selected DEPs. The top 5 biological processes were involving platelet degranulation, neutrophil degranulation, integrin cell surface interactions, and regulation of insulin‐like growth factor (IGF) transport and uptake by insulin‐like growth factor binding proteins (IGFBPs) (Figure 2H).

To explore the interaction relationships of DEPs, the top 25 proteins with the highest connectivity were selected, and protein‐protein interactions (PPI) were visualized in Figure 2I. Proteins upregulated in APS were represented in red, while downregulated proteins were shown in blue. The top proteins in terms of connectivity for both upregulated and downregulated proteins were shown in Figure 2J–M, including thrombospondin‐1 (TSP1, FC = 0.44, degree = 16), integrin beta‐3 (ITB3, FC = 0.10, degree = 14), matrix metalloproteinase‐9 (MMP9, FC = 2.10, degree = 18), and osteopontin (OSTP, FC = 2.39, degree = 15).

**Figure 3:**
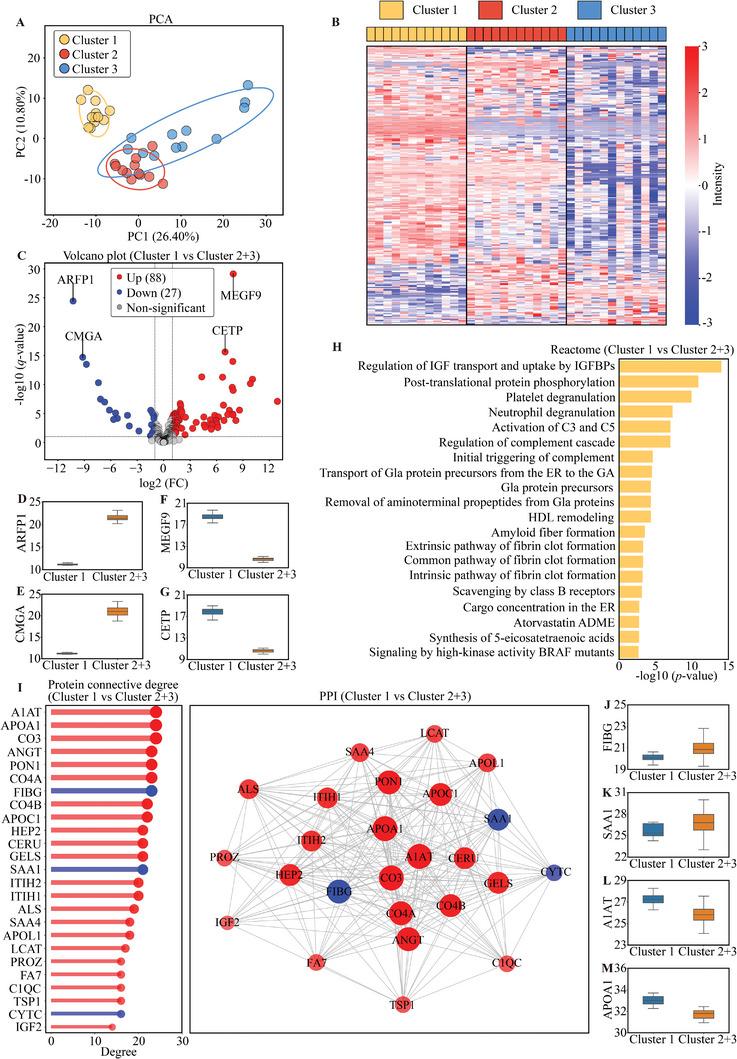
DEPs and bioinformatic analysis (Cluster 1 vs Cluster 2+3). A) PCA score plot for proteomic in Cluster 1 (*n* = 12) versus Cluster 2+3 (*n* = 24), the ellipse represents the 95% confidence interval. B) Heat map showing DEPs in Cluster 1 versus Cluster 2+3. C) Volcano plot showing DEPs in Cluster 1 versus Cluster 2+3. D–G) Box plots displaying the two most significantly downregulated (ARFP1, CMGA) and upregulated (MEGF9, CETP) proteins in Cluster 1 versus Cluster 2+3, selected based on the highest negative log10‐transformed *q*‐values. H) Top 20 Reactome pathway enrichment results of DEPs in Cluster 1 versus Cluster 2+3. I) The top 25 connection degree proteins and PPI network in Cluster 1 versus Cluster 2+3. Proteins upregulated in Cluster 1 were represented in red, while downregulated proteins were shown in blue. The size of the circle represented the magnitude of the connection. J–M) Box plots illustrating proteins with the highest connectivity degrees in Cluster 1 versus Cluster 2+3, showing FIBG and SAA1 as the most significantly downregulated and A1AT and APOA1 as the most upregulated.

The variance between Cluster 1 and Clusters 2+3 was performed by PCA analysis (**Figure** **3**A). By applying a *q*‐value threshold of less than 0.1 and considering proteins that exhibit at least two FCs, a total of 115 proteins were differentially expressed in Cluster 1 (88 upregulated, 27 downregulated) (Figure 3B). The key DEPs were visualized in volcano plot in Figure 3C, including arfaptin‐1 (ARFP1, FC = 8.03 × 10^−4^, *q*‐value = 3.79 × 10^−25^), chromogranin‐A (CMGA, FC = 1.71 × 10^−3^, *q*‐value = 1.97 × 10^−15^), multiple epidermal growth factor‐like domains protein 9 (MEGF9, FC = 239.13, *q*‐value = 7.70 × 10^−30^), and cholesteryl ester transfer protein (CETP, FC = 125.63, *q*‐value = 2.35 × 10^−16^), with their specific expression levels presented in Figure 3D–G. Reactome analysis of DEPs in Cluster 1 identified enrichment in 20 pathways compared with Clusters 2+3, chiefly comprising regulation of IGF transport and uptake by IGFBPs, post‐translational protein phosphorylation, platelet degranulation, and neutrophil degranulation (Figure 3H). The top 25 connection degree proteins and PPI network are shown in Figure 3I. The analysis identified fibrinogen gamma chain (FIBG, FC = 0.47, degree = 23), serum amyloid A‐1 protein (SAA1, FC = 0.37, degree = 21), alpha‐1‐antitrypsin (A1AT, FC = 2.86, degree = 24), and apolipoprotein A1 (APOA1, FC = 2.51, degree = 24) as the top two proteins with the highest connectivity in both upregulated and downregulated proteins, respectively (Figure 3J–M).

**Figure 4:**
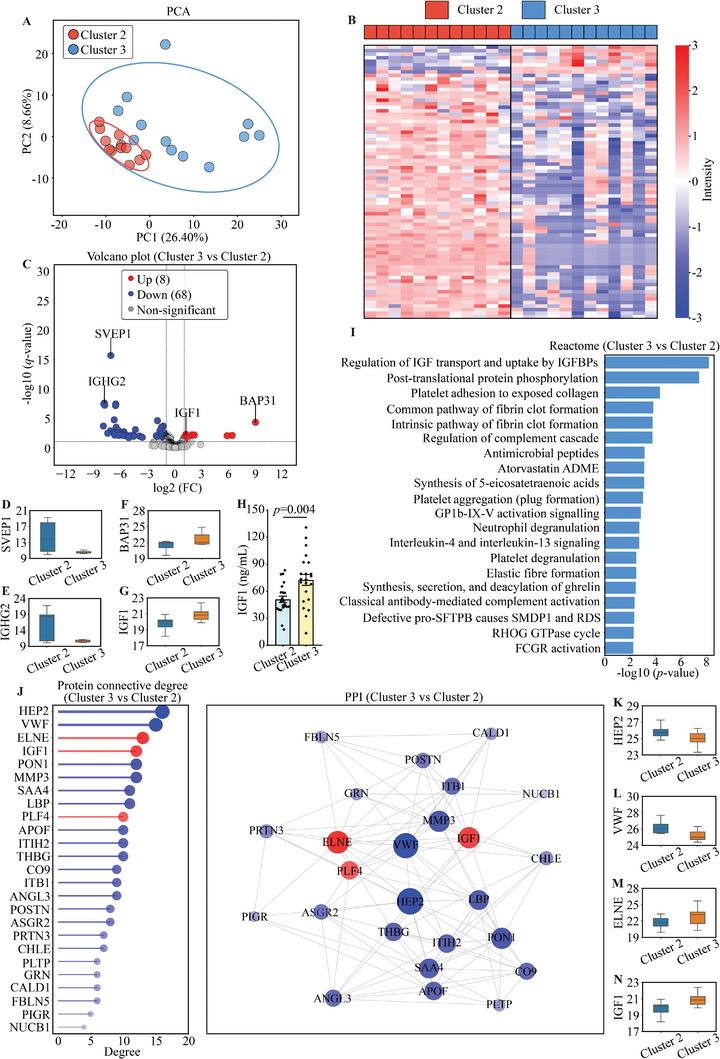
DEPs and bioinformatic analysis (Cluster 3 vs Cluster 2). A) PCA score plot for proteomic in Cluster 2 (*n* = 12) versus Cluster 3 (*n* = 12), the ellipse represents the 95% confidence interval. B) Heat map showing DEPs in Cluster 2 versus Cluster 3. C) Volcano plot showing DEPs in Cluster 3 versus Cluster 2. D–G) Box plots displaying the two most significantly downregulated (SVEP1, IGHG2) and upregulated (BAP31, IGF1) proteins in Cluster 3 versus Cluster 2, selected based on the highest negative log10‐transformed *q*‐values. H) ELISA validation of IGF1, *n* = 22 for Cluster 2 and Cluster 3, respectively. Statistical significance was tested using unpaired Student's *t*‐test. I) Top 20 Reactome pathway enrichment results of DEPs in Cluster 2 versus Cluster 3. J) The top 25 connection degree proteins and PPI network in Cluster 3 versus Cluster 2. Proteins upregulated in Cluster 3 were represented in red, while downregulated proteins were shown in blue. The size of the circle represented the magnitude of the connection. K–N) Box plots illustrating proteins with the highest connectivity degrees in Cluster 3 versus Cluster 2, showing HEP2 and VWF as the most significantly downregulated and ELNE and IGF1 as the most upregulated.

The proteomic profiles of Cluster 2 and Cluster 3 were differentiated by PCA analysis (**Figure** **4**A). By using a *q*‐value of less than 0.1 and considering proteins with at least two FCs, the analysis identified 76 DEPs in Cluster 3 compared to Cluster 2 (8 upregulated, 68 downregulated) (Figure 4B). The key DEPs were mapped by using a volcano plot (Figure 4C), including sushi, von Willebrand factor type A, EGF and pentraxin domain‐containing protein 1 (SVEP1, FC = 6.84 × 10^−3^, *q*‐value = 2.49 × 10^−16^), immunoglobulin heavy constant gamma 2 (IGHG2, FC = 4.17 × 10^−3^, *q*‐value = 2.92 × 10^−8^), B‐cell receptor‐associated protein 31 (BAP31, FC = 489.79, *q*‐value = 5.35 × 10^−5^), and IGF1 (FC = 2.27, *q*‐value = 6.06 × 10^−3^). The expression levels of these key proteins are shown in Figure 4D–G. The top 20 biological processes from Reactome analysis revealed notable enrichment in pathways for DEPs in Cluster 3, which mainly included regulation of IGF transport and uptake by IGFBPs, post‐translational protein phosphorylation, platelet adhesion to exposed collagen, and common pathway of fibrin clot formation (Figure 4H). The pathways were primarily enriched in the IGF pathway, with IGF1 identified as a key molecule. Therefore, we validated the IGF1 expression by using ELISA. We confirmed a higher IGF1 expression in Cluster 3 compared to Cluster 2 (Figure 4I). The protein connectivity and PPI network are presented in Figure 4J. The top two proteins with the highest connectivity were heparin cofactor 2 (HEP2, FC = 0.49, degree = 16), von Willebrand factor (VWF, FC = 0.49, degree = 15), neutrophil elastase (ELNE, FC = 2.76, degree = 13), and IGF1 (FC = 2.27, degree = 12) in the upregulated and downregulated proteins, respectively, as shown in Figure 4K–N.

## 3 Discussion

In the present, by applying an unsupervised K‐means cluster analysis in large‐scale multicenter cohorts, we identify three APS subgroups, i.e., oAPS, female tAPS, and male tAPS. We demonstrate that there are discernible differences between oAPS and tAPS. There are also distinct differences between male and female patients with tAPS. The male and female patients with tAPS display distinct clinical manifestations and molecular features, as well as a marked difference in prognostic outcomes. The male tAPS patients display a worse prognosis. We further identify that IGF1 is a crucial molecule linked to poor prognosis in male tAPS subgroup.

It has been reported that a relatively lower proportion of obstetric patients developed thrombosis.^[^ ^11^, ^19^, ^20^, ^21^, ^22^ ^]^ In the present study, we also observe that female APS patients with pregnancy morbidity (Cluster 1) or with thrombosis (Cluster 2) are clearly separated into two subgroups. Majority of the oAPS patients are primary APS (70.6%) with a low‐risk thrombosis, suggesting non‐thrombotic mechanisms may be more important in the pathogenesis of oAPS. Furthermore, previous studies report there is a high incidence of positivity to anti‐β2GPI^[^ ^23^ ^]^ or triple aPLs^[^ ^24^ ^]^ among APS patients with pregnancy morbidity. However, in this study we find that the oAPS group shows a lower positivity to single, double, or triple aPLs. In addition, consistent with previous findings, our oAPS patients also display the lowest incidence of complement reduction.^[^ ^25^ ^]^ Moreover, consistent with previous studies, we also observe that female APS patients with younger age at disease onset are more likely to present with pregnancy morbidity.^[^ ^26^ ^]^

In the present study, we observe an obvious sex difference between male and female patients with tAPS. The female tAPS group has an advanced onset (median age = 50) and relatively fewer primary APS patients (43.7%) with a poor prognosis. Nearly half of the patients complicate with thrombocytopenia, decreased complements, and metabolic syndrome, indicating female tAPS patients are more likely to be secondary APS. The higher incidence of metabolic syndrome in female tAPS patients may be attributed to multiple factors such as sex differences, an elder onset age, endocrine disorders, and mental health.^[^ ^27^, ^28^ ^]^ And a meta‐analysis suggests an independent association between metabolic syndrome and unprovoked venous thromboembolism.^[^ ^29^ ^]^ In contrast, the male tAPS group has a relatively younger onset (median age = 41) and more primary APS patients (58.8%). This group also displays the highest rates of positivity to LA (74.8%) and triple aPLs (31.3%). This group also has a higher number of smokers (19.1%) compared to the female tAPS group (7.1%). In addition, we find that the male tAPS patients has a poorest prognosis, which is consistent with previous study.^[^ ^30^ ^]^ It has been reported that smoking is associated with increased arterial events and a poor prognosis in tAPS patients.^[^ ^31^ ^]^ And majority of male APS patients suffer from arterial thrombosis and experience more recurrent events.^[^ ^32^ ^]^

In this study, we observe a clear difference in immunocyte phenotypes between three subgroups. A previous study has reported that immune cell dysregulation was linked to pathological pregnancy.^[^ ^33^ ^]^ We also observe that the treatment‐naïve oAPS patients from pregnancy morbidity group (Cluster 1) has more T, B, and NK cells than those thrombotic groups (Cluster 2 and Cluster 3), suggesting an excessive immune activation in oAPS patients. Notably, hydroxychloroquine could suppress T‐cell activation and cytokine production.^[^ ^34^ ^]^ Glucocorticoids play a crucial role in regulating immune activation and have a potent immunosuppressive effect on pro‐inflammatory T cells.^[^ ^35^ ^]^ Furthermore, intravenous immunoglobulin (IVIG) therapy modulates multiple NK cell and T cell functions in patients with immune dysregulation.^[^ ^36^ ^]^ Thus, the remarkable increases in T, B, and NK cells in Cluster 1 may shed light on the effective use of hydroxychloroquine, corticosteroids, and IVIG for oAPS patients in clinical practice. Additionally, other studies have reported that levels of IFN‐γ are elevated during thrombosis,^[^ ^37^ ^]^ and Th1 and Th17 cells are increased in patients with SLE,^[^ ^38^ ^]^ or chronic immune thrombocytopenia.^[^ ^39^ ^]^ These studies are consistent with our observation that the patients in Cluster 2 are mostly female thrombotic APS, with the highest incidence of secondary APS and thrombocytopenia and the highest number of IFN‐γ producing Th1 cells and IL‐17 producing Th17 cells. However, it still remains elusive how the Th1/Th17 cells participate in the pathogenesis of thrombosis in APS. Notably, it is currently known that the neutrophil extracellular traps (NETs) could induce naïve CD4^+^ T cell differentiating into a Th17 phenotype,^[^ ^40^ ^]^ and the NETosis is recognized as an important event involved in the pathogenesis of SLE and APS, especially in thrombotic APS.^[^ ^41^ ^]^ Further mechanistic studies are warranted to elucidate the complex interplay between NETosis and Th17 cells contributing to the thrombotic APS. Furthermore, previous research indicates that the occurrence of thrombosis increased number of Tregs,^[^ ^42^ ^]^ which consistent with our findings that Cluster 3 has the highest number of thrombotic events and also shows an increased numbers of Tregs and IL‐2 producing CD4^+^ T cells.

Proteomic analysis also reveals a distinct molecular feature between three APS subgroups and identified IGF1 as a crucial molecule linked to male tAPS. IGF1 is a 70‐amino acid basic peptide hormone that is expressed in most tissues. IGF1 levels are associated with an increased risk of cardiovascular disease. This association is predominant in males but not in females.^[^ ^43^ ^]^ IGF1 expression is significantly increased in Cluster 3, which is characterized by male patients with thrombosis. IGF1 enhances platelet Akt phosphorylation and activity, eventually inducing thrombosis in murine model of type I diabetes.^[^ ^44^ ^]^ Anti‐β2GPI antibodies could also activate platelet Akt pathway to promote thrombosis in APS patients.^[^ ^45^, ^46^ ^]^ Targeting IGF1 pathway may provide a novel therapeutic approach for male patients with tAPS.

Our study has limitations. First, as the laboratory data could not be mutually recognized between different hospitals. Therefore, some of the results can only be converted into qualitative data for analysis. Second, secondary APS is not excluded from this study.

In conclusion, in this work, we identify three distinct APS subgroups, i.e., female oAPS, female tAPS, and male tAPS. Each subgroup display unique clinical manifestations and molecular features, as well as a marked difference in prognostic outcomes.

## 4 Experimental Section

### Study Participants and Ethics Review

This is a multicenter cohort study in patients with APS. All patients fulfilled the 2006 Sapporo criteria^[^ ^47^ ^]^ and/or the 2023 ACR/EULAR APS classification criteria.^[^ ^48^ ^]^

A two‐stage (training and validation) study was conducted with a total of 760 APS patients enrolled (training cohort: *n* = 415, validation cohort: *n* = 345). The validation cohort was consisted of two sub‐cohorts, including an internal sub‐cohort (*n* = 238) and an external sub‐cohort (*n* = 107). The external validation sub‐cohort included patients from other three Grade A tertiary hospitals, including Peking University Third Hospital (*n* = 97), Beijing Chaoyang Hospital Affiliated to Capital Medical University (*n* = 5), and Xinxiang Central Hospital (*n* = 5).

This study was approved by the ethics committees of Peking University People's Hospital (No. 2024PHB041‐001) and implemented according to the principles of the Declaration of Helsinki Good Clinical Practice guidelines.

### Data Collection and Variable Definitions

A total of six variables were included and collected retrospectively at the time of the registry. These variables covered a broad range of demographic, clinical, and laboratory indexes, including age, sex, pregnancy morbidity, thrombotic events, other clinical indicators, and a collection of abnormal hematological and serological indicators, including thrombocytopenia, hemolytic anemia, immunoglobulin, and complement abnormalities. In which other clinical indicators were also combined as one single variable except for the typical clinical events, i.e., pregnancy morbidity and thrombotic events (detailed in Table S1, Supporting Information). Diagnosis of the pregnancy morbidity and thrombotic events were according to the 2006 Sapporo criteria and/or the 2023 ACR/EULAR APS classification criteria. Hypertension was classified according to the 2018 ESC/ESH guidelines.^[^ ^49^ ^]^ Hyperlipidemia was defined by the 2019 ACC/AHA guidelines.^[^ ^50^ ^]^ Diabetes was defined according to the 1999 WHO standards.^[^ ^51^ ^]^ Psychiatric symptoms were defined according to International Classification of Diseases, 10th Revision (ICD‐10) codes, including depression, anxiety, schizophrenia, bipolar disorders, and cognitive deficit.^[^ ^52^ ^]^ Thrombocytopenia was defined as a platelet count of less than 100×10^9^/L at least twice with 12 weeks apart.^[^ ^47^ ^]^ The following thrombocytopenic conditions were excluded from this study, including (1) thrombotic thrombocytopenic purpura, (2) disseminated intravascular coagulation, (3) pseudo‐thrombocytopenia, (4) heparin‐induced thrombocytopenia or other drug‐induced thrombocytopenia, (5) infection, (6) and hematologic disorders. The total immunoglobulins (IgG, IgA, IgM) were applied for analysis. They were quantified by using the nephelometry assay. The different clusters were then evaluated for their prognostic outcomes, including recurrent thrombosis, major bleeding, and mortality. Major bleeding occured in critical areas or organs, such as intracranial bleeding (confirmed by imaging), retroperitoneal bleeding, intraspinal bleeding, intraocular bleeding leading to blindness, pericardial bleeding, joint hemorrhage, or situations necessitating surgical or angiographic intervention to control the hemorrhage.

### Model Development and Endotype Discovery

In the present study, a linear PCA was performed to reduce the dimensionality of the original data into two principal components. By extracting the two principal components, which accounted for majority of the variance, it was aimed to minimize the loss of information during the process of capturing key variables.^[^ ^53^ ^]^ A total of six variables were applied for the PCA, as detailed in “Data Collection and Variable Definitions” and Table S1 (Supporting Information). After dimensionality reduction, the K‐means, an unsupervised machine learning algorithm, was employed to discern intrinsic groupings within the dataset. Observations were systematically assigned to the nearest cluster centroid, measured by Euclidean distance, ensuring precise subgroup delineation.^[^ ^54^ ^]^ The optimal numbers for the K‐means clustering (K) were defined within a range of 2–5 based on silhouette coefficient. The optimal K coefficient identified in training cohort was further validated in both internal and external cohorts.

### Proteomic Analysis

Proteomic analysis was performed on treatment‐naïve patients with primary APS (pAPS) from three identified clusters (*n* = 36, 12 from each cluster) and HCs (*n* = 12). The clinical characteristics of the selected APS patients were shown in Table S2 (Supporting Information). Bicinchoninic acid (BCA) assay was applied for protein quantitation. 500 µg protein was loaded onto High‐Select TM Top14 Abundant Protein Depletion Mini Spin Columns (Thermo Fisher Scientific, USA) to enrich the low‐abundance proteins. The filtrate was exchanged into 50 mm ABC buffer, reduced, and alkylated, followed by overnight tryptic digestion at 37 °C. The digestion was terminated by adding 0.1% formic acid, and the resulting peptides were desalted using C18 spin column and dried using a speed‐vac. The peptides were reconstituted in 50 µL of mobile phase A before LC‐MS/MS. Data acquisition was achieved through data‐independent acquisition methods established by Pino et al.^[^ ^55^ ^]^ The PCA analysis was used the “scikit‐learn” package in Python 3.10. Data were log2‐transformed, and DEPs were filtered based on a *q*‐value of less than 0.1 and at least two FCs. Heatmaps and volcano plots were created using the “seaborn” and “matplotlib” packages of Python 3.10 to visually display the expression changes of DEPs. Pathway enrichment analysis was performed using the Reactome database (https://reactome.org/), and the 20 pathways with the lowest *p* values were visualized using “matplotlib” packages in Python 3.10. PPI among the DEPs were analyzed using data from the STRING database (https://string‐db.org/). A network of the top 25 proteins with the highest connectivity was plotted, and a bar chart showing their connectivity was generated using the “matplotlib” and “network” packages in Python 3.10.

### Enzyme‐linked Immunosorbent Assay

Serum IGF1 levels were measured using IGF1 ELISA kit (DRG Instruments GmbH, Marburg, Germany). ELISA was performed according to the manufacturers protocol.

### Statistical Analysis

In this study, to minimize interference of different cut‐off values for indicators across hospitals, all indicators were converted into qualitative variables for clustering analysis except for age. Only a few specific numerical values were applied during detailed analysis. All datasets for the clustering analysis were standardized using z‐score normalization, and all data for the indicators included in clustering were analyzed. Sample size (*n*) for each statistical analysis was presented in Table S3 (Supporting Information). Categorical variable data were expressed as *n* (%). The Chi‐square test was used for comparing proportions among different groups. Normal distribution was determined using the Shapiro‐Wilk test. Normally distributed continuous variables were expressed as means ± standard deviations and compared using the unpaired Student's *t*‐test or one‐way ANOVA. Non‐normal continuous variables were indicated as median and IQR (25th ‐75th percentiles). The Mann‐Whitney *U* test or Kruskal‐Wallis test was implemented to compare distinct groups. Survival analyses were performed using Kaplan‐Meier analyses and compared using the log‐rank test. All tests were two‐sided, with *p* < 0.05 regarded as statistically significant. All statistical analyses were performed using Python 3.10. *p* values less than 0.05 were considered as statistically significant.

### Ethics Approval

The study protocols were approved by the Institutional Medical Ethics Review Board of Peking University People's Hospital (No. 2024PHB041‐001).

## Conflict of Interest

The authors declare no conflict of interest.

## Author Contributions

C.C. and A.Z. contributed equally as first authors. C.L. and J.G. performed project administration and supervision; C.L., J.G., F.X., and X.L. performed conceptualization; C.C. performed formal analysis; C.C. and C.L. wrote the original draft; C.L., J.G., C.C., A.Z., and J.C. wrote, reviewed and edited the manuscript; C.C. and A.Z. performed visualization and validation; C.L., J.G., Z.Y., J.M., and Y.Q. collected resources; C.C., Q.L., Y.L., and X.L. performed data curation; C.C., A.Z., T.L., and C.H. performed software; J.C., Y.T., H.L., N.X., and S.D. performed investigation and methodology. All authors reviewed the manuscript and approved the final version submitted for publication. The Corresponding Authors have the right to grant on behalf of all authors and do grant on behalf of all authors.

## Supporting information



Supporting Information

## Data Availability

The data underlying this article cannot be shared publicly due to the privacy of individuals that participated in the study. The data will be shared on reasonable request to the corresponding author.
