# Supplementary material for: Identification of Three Distinct Subgroups in Antiphospholipid Syndrome: Implication for Sex Differences and Prognostic Outcomes from a Multicenter Study
Source: Adv Sci (Weinh). 2025 Feb 18;12(15):2415291. doi: 10.1002/advs.202415291 (PMC12005735; doi:10.1002/advs.202415291)
Supplement: Supplementary file 1 — Supporting Information [file ADVS-12-2415291-s001.pdf]

ADVANCED SCIENCE
Open Access

Supporting Information

for *Adv. Sci.*, DOI 10.1002/advs.202415291

Identification of Three Distinct Subgroups in Antiphospholipid Syndrome: Implication for Sex Differences and Prognostic Outcomes from a Multicenter Study

Chen Chen, Ao Zhang, Jianhui Cheng, Zhongqiang Yao, Juan Meng, Yilu Qin, Qingyi Lu, Yufei Li, Xiangjun Liu, Tianhao Li, Chao Hou, Yundi Tang, Hongjiang Liu, Ning Xu, Sai Dong, Xinxin Li, Fangmin Xu, Jianping Guo and Chun Li**

SUPPLEMENTARY MATERIAL

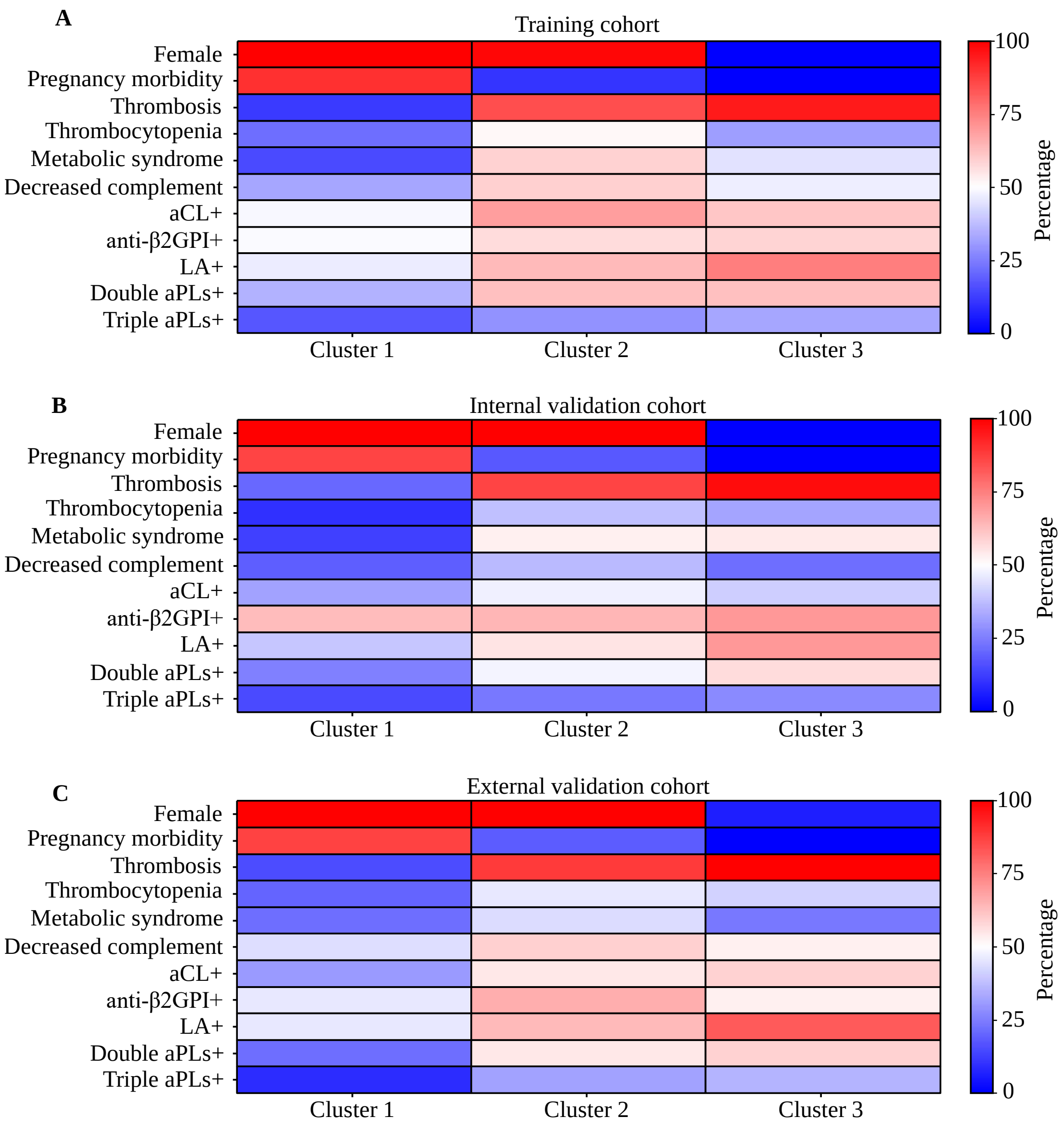

Supplementary Figure 1. Main characteristics in three cohorts. Heat maps showing similar varying trend of main characteristics: **A)** in training cohort (n=415). **B)** in internal validation cohort (n=238). **C)** in external validation cohort (n=107).

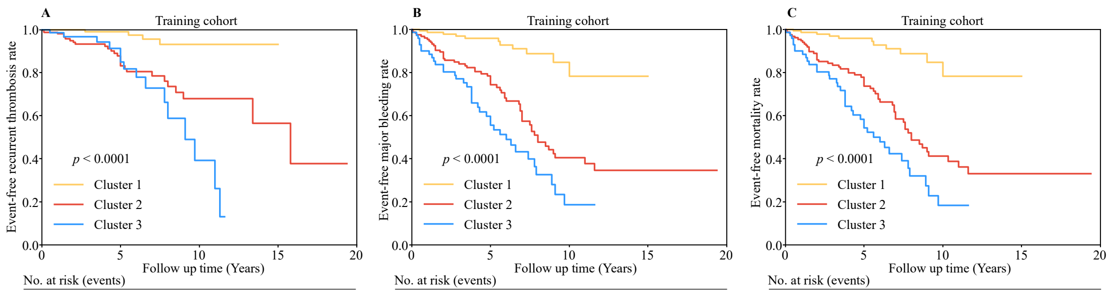

No. at risk (events)

Years	0	5	10	15	20
Cluster 1	171(0)	75(1)	13(3)	1(0)	0(0)
Cluster 2	167(0)	75(18)	17(7)	3(1)	0(1)
Cluster 3	77(0)	29(6)	4(7)	0(2)	0(0)

No. at risk (events)

Years	0	5	10	15	20
Cluster 1	171(0)	75(5)	13(6)	1(0)	0(0)
Cluster 2	167(0)	75(32)	17(22)	3(2)	0(0)
Cluster 3	77(0)	29(26)	4(11)	0(0)	0(0)

No. at risk (events)

Years	0	5	10	15	20
Cluster 1	171(0)	75(5)	13(6)	1(0)	0(0)
Cluster 2	167(0)	75(33)	17(21)	3(3)	0(0)
Cluster 3	77(0)	29(27)	4(11)	0(0)	0(0)

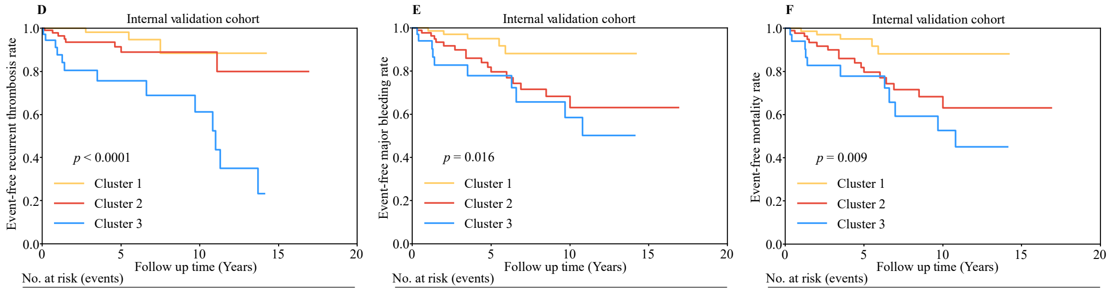

No. at risk (events)

Years	0	5	10	15	20
Cluster 1	103(0)	31(1)	3(2)	0(0)	0(0)
Cluster 2	98(0)	38(7)	13(0)	1(1)	0(0)
Cluster 3	37(0)	15(7)	8(2)	0(4)	0(0)

No. at risk (events)

Years	0	5	10	15	20
Cluster 1	103(0)	31(3)	3(2)	0(0)	0(0)
Cluster 2	98(0)	38(12)	13(5)	1(0)	0(0)
Cluster 3	37(0)	15(6)	8(3)	0(1)	0(0)

No. at risk (events)

Years	0	5	10	15	20
Cluster 1	103(0)	31(3)	3(2)	0(0)	0(0)
Cluster 2	98(0)	38(12)	13(5)	1(0)	0(0)
Cluster 3	37(0)	15(6)	8(4)	0(1)	0(0)

Supplementary Figure 2. Cumulative incidence of event-free survival. Kaplan–Meier analysis of cumulative event-free survival in APS patients, showing recurrent thrombosis, major bleeding, and mortality event-free survival: **A-C)** in training cohort (n=415). **D-F)** in internal validation cohort (n=238). The numbers below the figures denoted the number of patients at risk in each cluster. The *p* values were calculated with log-rank test.

Supplementary Table 1. Variables included in the cluster analysis

Category	Variable
Age	Age at diagnosis (years)
Sex	Male; Female
Pregnancy morbidity	According to the 2006 Sapporo criteria and/or the 2023 ACR/EULAR APS classification criteria
Thrombotic events	According to the 2006 Sapporo criteria and/or the 2023 ACR/EULAR APS classification criteria
Other clinical indicators (at least one of the following)	Hypertension; Hyperlipidemia; Diabetes; CHD; Psychiatric symptoms; Arrhythmias; Cardiac hypertrophy; Cardiac valve disease; Heart failure; Alveolar hemorrhage; Digital gangrene; Lower limb skin ulcer; Livedo reticularis; Subcutaneous nodule
Thrombocytopenia, hemolytic anemia, immunoglobulin and complement abnormalities (at least one of the following)	Thrombocytopenia; Hemolytic anemia; Elevated IgA; Elevated IgG; Elevated IgM; Decreased C3 levels; Decreased C4 levels

CHD, coronary heart disease; Ig, immunoglobulin; C3, complement 3; C4, complement 4.

Supplementary Table 2. Clinical characteristics of three APS clusters and HCs for proteomics

	Cluster 1 (n=12)	Cluster 2 (n=12)	Cluster 3 (n=12)	HC (n=12)
Female, n (%)	12 (100%)	12 (100%)	0 (0%)	7 (58.3%)
Age, years, median (IQR)	34 (33-38)	60 (51-63)	45 (33-54)	43 (35-54)
Pregnancy morbidity, n (%)	12 (100%)	0 (0%)	0 (0%)	--
Thrombosis, n (%)	0 (0%)	12 (100%)	12 (100%)	--
Arterial thrombosis, n (%)	0 (0%)	7 (58.3%)	5 (41.7%)	--
Venous thrombosis, n (%)	0 (0%)	8 (66.7%)	8 (66.7%)	--
Thrombocytopenia, n (%)	2 (16.7%)	7 (58.3%)	5 (41.7%)	--
Hemolytic anemia, n (%)	0 (0%)	2 (16.7%)	1 (8.3%)	--
Neuropsychiatric disorders, n (%)	0 (0%)	4 (33.3%)	0 (0%)	--
Smoking, n (%)	1 (8.3%)	3 (25.0%)	5 (41.7%)	--
Metabolic syndrome, n (%)	1 (8.3%)	8 (66.7%)	6 (50.0%)	--
Hypertension, n (%)	1 (8.3%)	6 (50.0%)	2 (16.7%)	--
Hyperlipidemia, n (%)	0 (0%)	3 (25.0%)	2 (16.7%)	--
Diabetes, n (%)	1 (8.3%)	4 (33.3%)	5 (41.7%)	--

Supplementary Table 3. Sample size (n) for each statistical analysis

	Cluster 1 (n)	Cluster 2 (n)	Cluster 3 (n)	HC (n)
Clustering	320	309	131	--
Training cohort	171	167	77	--
Internal validation cohort	103	98	37	--
External validation cohort	46	44	17	--
Clinical and laboratory characteristics	320	309	131	--
Training cohort	171	167	77	--
Internal validation cohort	103	98	37	--
External validation cohort	46	44	17	--
Immunological characteristics (naïve APS patients)	--	--	--	--
Total lymphocytes	46	12	11	--
$CD19^{+}$B cells	46	12	11	--
$CD3^{+}$T cells	46	12	11	--
$CD4^{+}$T cells	46	12	11	--
$CD8^{+}$T cells	46	12	11	--
$CD3^{-}CD16^{+}$ and/or $CD56^{+}$ NK cells	46	12	11	--
$CD4^{+}CD25^{-/+}Foxp3^{-}$ Teffs	46	12	9	--
$CD4^{+}CD25^{hi}Foxp3^{+}$ Tregs	46	12	9	--
IL-17 producing Th17 cells	22	7	7	--
IFN-γ producing Th1 cells	22	7	7	--
IL-2 producing $CD4^{+}$ T cells	22	7	7	--
Survival analyses	274	265	114	--
Training cohort	171	167	77	--
Internal validation cohort	103	98	37	--
Proteomic analysis	12	12	12	12
Enzyme-linked immunosorbent assay	22	22	22	14

Supplementary Table 4. Clinical characteristics of three APS cohorts

	Training cohort (n=415)				Internal validation cohort (n=238)				External validation cohort (n=107)			
	Cluster 1 (n=171, 41.2%)	Cluster 2 (n=167, 40.2%)	Cluster 3 (n=77, 18.6%)	*p*-value	Cluster 1 (n=103, 43.3%)	Cluster 2 (n=98, 41.2%)	Cluster 3 (n=37, 15.5%)	*p*-value	Cluster 1 (n=46, 43.0%)	Cluster 2 (n=44, 41.1%)	Cluster 3 (n=17, 15.9%)	*p*-value
Female, n (%)	171 (100%)	165 (98.8%)	0 (0%)	<0.001	103 (100%)	98 (100%)	0 (0%)	<0.001	46 (100%)	52 (98.1%)	1 (5.9%)	<0.001
Age, years, median (IQR)	34 (31-38)	53 (39-63)	41 (32-57)	<0.001	33 (31-38)	44 (34-62)	49 (34-57)	<0.001	32 (29-36)	49 (36-57)	34 (26-39)	<0.001
aGAPSS, median (IQR)	5 (4-9)	9 (7-13)	9 (5-13)	<0.001	4 (4-8)	8 (4-13)	8 (5-13)	<0.001	4 (4-8)	9 (4-13)	9 (4-13)	0.002
Pregnancy morbidity, n (%)	155 (90.6%)	17 (10.2%)	0 (0%)	<0.001	89 (86.4%)	17 (17.3%)	0 (0%)	<0.001	40 (87.0%)	8 (18.2%)	0 (0%)	<0.001
Thrombosis, n (%)	20 (11.7%)	141 (84.4%)	73 (94.8%)	<0.001	21 (20.4%)	85 (86.7%)	36 (97.3%)	<0.001	7 (15.2%)	39 (88.6%)	17 (100%)	<0.001
Arterial thrombosis, n (%)	8 (4.7%)	87 (52.1%)	37 (48.1%)	<0.001	12 (11.7%)	45 (45.9%)	25 (67.6%)	<0.001	5 (10.9%)	25 (56.8%)	11 (64.7%)	<0.001
Venous thrombosis, n (%)	14 (8.2%)	83 (49.7%)	46 (59.7%)	<0.001	13 (12.6%)	54 (55.1%)	17 (45.9%)	<0.001	3 (6.5%)	19 (43.2%)	10 (58.8%)	<0.001
Thrombocytopenia, n (%)	37 (21.6%)	86 (51.5%)	24 (31.2%)	<0.001	10 (9.7%)	37 (37.8%)	12 (32.4%)	<0.001	12 (26.1%)	21 (47.7%)	7 (41.2%)	0.202
Hemolytic anemia (HA), n (%)	8 (4.7%)	28 (16.8%)	5 (6.5%)	0.002	3 (2.9%)	15 (15.3%)	3 (8.1%)	0.022	0 (0%)	2 (4.5%)	1 (5.9%)	0.492
Neuropsychiatric disorders, n (%)	0 (0%)	24 (14.4%)	5 (6.5%)	<0.001	0 (0%)	9 (9.2%)	1 (2.7%)	0.013	2 (4.3%)	9 (20.5%)	3 (17.6%)	0.139
Smoking, n (%)	17 (9.9%)	15 (9.0%)	14 (18.2%)	0.178	1 (1.0%)	1 (1.0%)	7 (18.9%)	<0.001	0 (0%)	6 (13.6%)	4 (23.5%)	0.021
Primary APS, n (%)	112 (65.5%)	72 (43.1%)	43 (55.8%)	<0.001	81 (78.6%)	43 (43.9%)	25 (67.6%)	<0.001	33 (71.7%)	20 (45.5%)	9 (52.9%)	0.086
Metabolic syndrome, n (%)	25 (14.6%)	98 (58.7%)	34 (44.2%)	<0.001	13 (12.6%)	52 (53.1%)	17 (45.9%)	<0.001	10 (21.7%)	19 (43.2%)	4 (23.5%)	0.148
Hypertension, n (%)	14 (8.2%)	67 (40.1%)	23 (29.9%)	<0.001	9 (8.7%)	41 (41.8%)	14 (37.8%)	<0.001	8 (17.4%)	14 (31.8%)	3 (17.6%)	0.394
Hyperlipidemia, n (%)	9 (5.3%)	46 (27.5%)	15 (19.5%)	<0.001	2 (1.9%)	23 (23.5%)	6 (16.2%)	<0.001	4 (8.7%)	7 (15.9%)	4 (23.5%)	0.478
Diabetes, n (%)	10 (5.8%)	28 (16.8%)	8 (10.4%)	0.016	7 (6.8%)	15 (15.3%)	4 (10.8%)	0.281	1 (2.2%)	7 (15.9%)	1 (5.9%)	0.129

IQR, interquartile range; aGAPSS, adjusted global anti-phospholipid syndrome score. Significance was assessed using one-way ANOVA.

Supplementary Table 5. Laboratory characteristics of three APS cohorts

	Training cohort (n=415)				Internal validation cohort (n=238)				External validation cohort (n=107)			
	Cluster 1 (n=171, 41.2%)	Cluster 2 (n=167, 40.2%)	Cluster 3 (n=77, 18.6%)	*p*-value	Cluster 1 (n=103, 43.3%)	Cluster 2 (n=98, 41.2%)	Cluster 3 (n=37, 15.5%)	*p*-value	Cluster 1 (n=46, 43.0%)	Cluster 2 (n=44, 41.1%)	Cluster 3 (n=17, 15.9%)	*p*-value
aCL positivity, n (%)	83 (48.5%)	115 (68.9%)	47 (61.0%)	0.002	33 (32.0%)	46 (46.9%)	15 (40.5%)	0.196	14 (30.4%)	24 (54.5%)	10 (58.8%)	0.076
anti-β2GPI positivity, n (%)	84 (49.1%)	95 (56.9%)	45 (58.4%)	0.422	65 (63.1%)	63 (64.3%)	26 (70.3%)	0.891	21 (45.7%)	29 (65.9%)	9 (52.9%)	0.287
LA positivity, n (%)	79 (46.2%)	106 (63.5%)	58 (75.3%)	<0.001	40 (38.8%)	54 (55.1%)	26 (70.3%)	0.007	21 (45.7%)	28 (63.6%)	14 (82.4%)	0.055
aCL and anti-β2GPI positivity, n (%)	38 (22.2%)	76 (45.5%)	35 (45.5%)	<0.001	21 (20.4%)	36 (36.7%)	14 (37.8%)	0.051	10 (21.7%)	20 (45.5%)	6 (35.3%)	0.128
aCL and LA positivity, n (%)	43 (25.1%)	68 (40.7%)	33 (42.9%)	0.008	18 (17.5%)	27 (27.6%)	10 (27.0%)	0.355	4 (8.7%)	16 (36.4%)	8 (47.1%)	0.004
anti-β2GPI and LA positivity, n (%)	37 (21.6%)	56 (33.5%)	30 (39.0%)	0.022	17 (16.5%)	30 (30.6%)	17 (45.9%)	0.004	4 (8.7%)	16 (36.4%)	8 (47.1%)	0.004
Double aPLs positivity, n (%)	60 (35.1%)	104 (62.3%)	48 (62.3%)	<0.001	26 (25.2%)	47 (48.0%)	21 (56.8%)	<0.001	10 (21.7%)	24 (54.5%)	10 (58.8%)	0.006
Triple aPLs positivity, n (%)	29 (17.0%)	48 (28.7%)	25 (32.5%)	0.023	15 (14.6%)	23 (23.5%)	10 (27.0%)	0.289	4 (8.7%)	14 (31.8%)	6 (35.3%)	0.032
Decreased complement, n (%)	56 (32.7%)	99 (59.3%)	36 (46.8%)	<0.001	19 (18.4%)	36 (36.7%)	8 (21.6%)	0.027	20 (43.5%)	26 (59.1%)	9 (52.9%)	0.529
Elevated Ig, n (%)	51 (29.8%)	76 (45.5%)	33 (42.9%)	0.023	19 (18.4%)	28 (28.6%)	9 (24.3%)	0.411	9 (19.6%)	14 (31.8%)	6 (35.3%)	0.495

aPL, antiphospholipid antibody; LA, lupus anticoagulant; aCL, anticardiolipin antibody; anti-β2GPI, anti-β2 glycoprotein I antibody; C3, complement 3; C4, complement 4; Ig, immunoglobulin. Significance was assessed using one-way ANOVA.
